# Cancer of unknown primary: Registered procedures compared with national integrated cancer pathway for illuminating external validity

**DOI:** 10.1097/MD.0000000000006693

**Published:** 2017-04-21

**Authors:** Anne-Kirstine Dyrvig, Knud Bonnet Yderstræde, Oke Gerke, Peter Bjødstrup Jensen, Søren Hess, Poul Flemming Høilund-Carlsen, Anders Green

**Affiliations:** a Centre for Innovative Medical Technology, Odense C; b Department of Surgery, Svendborg; c Department of Endocrinology; d Department of Nuclear Medicine, Odense University Hospital, Odense C; e Centre of Health Economics Research, University of Southern Denmark, Odense M; f Odense Patient data Explorative Network (OPEN), Odense University Hospital; g Department of Clinical Research, University of Southern Denmark, Odense C; h Department of Radiology and Nuclear Medicine, Hospital South West Jutland, Esbjerg, Denmark.

**Keywords:** clinical guideline, CUP, external validity, occult cancer, unknown primary

## Abstract

Cancer of unknown primary (CUP) ranges within top 10 cancers in both incidence and mortality. As primary identification is crucial to choosing treatment, guidelines on CUP emphasize the diagnostic strategy. Whether guidelines are complied with, or if they are indeed helpful, is however unclear. We compared procedures performed in suspected CUP patients with recommendations of national guidelines to assess external validity of guidelines.

The Danish National Patient Registry (NPR) comprising population data was utilized to identify the suspected CUP patients during 2009 to 2010 and explore exposure to procedures and patient survival. The cohort was investigated in terms of validity of diagnosis through cross-referencing with the Cancer Registry (CR), which served as gold standard for cancer diagnoses and patients’ cancer histories.

The NPR cohort consisted of 542 patients (275 males, 264 females) of whom 210 (38.7%) had a CUP diagnosis confirmed. Within the cohort, 347 patients (64.0%) had a registration in CR matching with the NPR registration. Exposure to diagnostic procedures included biopsy (n = 439, 81.0%) and image modalities (n = 532, 98.2%). Survival was poor with 67 (12.4%) individuals alive after 4 years.

The validity of a CUP diagnosis in NPR was low when using data from CR as reference. More than half the suspected CUP patients had a previous cancer diagnosis with CUP being the most frequent. Patients were diagnosed in compliance with guidelines indicating high external validity, but less than 1 quarter had their primary identified and the 1-year survival was approximately 20%. Research is needed to develop efficacious methods for primary detection.

## Abstract 2

Supplemental Digital Content is available in the text

## 1 Introduction

Cancer of unknown primary (CUP) accounts for 3% to 5% of all cancer malignancies,^[1–3]^ placing it among the top 10 cancers in terms of incidence and mortality among men and women.^[4,5]^ The prognosis is poor and severe morbidity often coexists at diagnosis, and, thus, being diagnosed with CUP is highly taxing on patients.^[6–9]^ Since identification of the primary is crucial to treatment triage and prognosis,^[10]^ an effective diagnostic strategy must be initiated when CUP is suspected. Not even postmortem investigation will necessarily lead to identification of the primary.^[11]^ In lieu of evidence from meta-analyses or systematic literature reviews, the best available evidence is constituted by clinical guidelines. To what degree diagnostic investigations comply with guidelines, or if such compliance is indeed helpful to patients, is not clear. One recommended approach for assessment of external validity is to compare results of randomized controlled trials (measuring efficacy) with results of cohort studies of the same intervention (measuring effectiveness) using routine data.^[12,13]^ In this study, registered procedures carried out in the routine in this type of patients were compared with recommendations of clinical guidelines to assess the external validity of the guidelines.

Denmark has the dubious honour of holding the world record on cancer incidence with 338 new cases per 100,000 in 2012, compared with 273 in the UK, 284 in Germany, 296 in Canada, and 318 in the United States.^[14]^ In 2007, this position in combination with a cancer death rate higher than in surrounding countries, prompted the National Danish Health Authority to establish patient pathways for diagnostics and treatment. By January 2009, these integrated cancer pathways were officially implemented in the Danish healthcare system. The national pathway for CUP constituted the reference guideline used as theoretical foundation for this article.^[15]^ The terms guideline and pathway are used interchangeably in the remainder of the article. Using 3 national databases, the aim of the present study was to determine the validity of registered CUP diagnoses, describe patients’ cancer history prior to the CUP diagnosis, assess if the clinical guideline for CUP had been complied with, and analyze survival following a CUP diagnosis with and without censoring for subsequent specific cancers.

## 2 Materials and methods

### 2.1 Data registries

Due to the comprehensive registration of each contact between a patient and the health care sector in Denmark, it is possible to study relatively large cohorts of patients and their history pre and post entry into a trial cohort. Further, thanks to personal ID numbers and free access to healthcare, 98% of which is public, Danish registries are not hampered by selection bias determined by access. We obtained pseudo-anonymised data from 3 registries: the National Patient Register (NPR), the Cancer Register (CR), and the national Civil Registration System (CRS); for details, see Supplemental Digital Content 1.

In NPR, a primary diagnosis must be registered at any admission. When a patient is discharged, the admission is ascribed to the most likely diagnosis at the time of discharge. Registrations in CR, on the other hand, are recorded alongside information on what clinical investigations confirm the presence of cancer.^[16]^ Consequently, registrations in CR reflect the knowledge at a later time point, when the result of a more decisive diagnostic workup is available, and divergences between registries are to be expected. In 2012, CR underwent thorough validation within the fields of lung and breast cancer (based on data from 2006). This validation included assessments of accuracy and completeness of the register with cases of disagreement explored through identification of the original data (i.e., patient files, pathology reports, etc.). The conclusion was that the register was valid with low risk of wrong or missing data.^[16]^ Assuming that the register is equally well managed within the field of CUP, CR was considered the more valid source of information compared to NPR, and cancer diagnoses in CR were considered true, whereas diagnoses in NPR were perceived as tentative. This assumption is related to the data validity rather than to the validity of the diagnosis.

The combination of NPR and CR registrations with the CRS data enabled both a validation of NPR registrations and analyses of CUP-patients’ diagnostic processes and cancer histories.

The CR data were not restricted to the 2009 to 2010 observation period. The follow-up for the cohort ended at the date of data request, namely August 1, 2013.

Permission to store the data was obtained by the Danish Data Protection Agency. No further permissions were required as registry studies do not require permission from the Regional Committee of Health Research Ethics for Southern Denmark.

### 2.2 CUP guideline

The authors of the national guideline state that it is based on evidence, previous clinical guidelines, or expert consensus.^[15]^ Specifically, 3 references are mentioned as the basis for the CUP guideline: a clinical guideline published by the Co-operative Cancer Departments,^[17]^ a clinical guideline published by the Danish Association for Head and Neck Oncology,^[18]^ and the clinical guideline published by Briasoulis et al^[19]^ in 2005. In short, the recommendations given in the national guideline were that patients suspected of CUP should be investigated through medical history, clinical investigation, blood sampling, image modalities, and biopsy.

Although the specific use of the scientific publications supporting these recommendations was not clear, the outline of steps within the diagnostic process facilitated explorations of whether or not the recommendations were complied with.

### 2.3 Statistical evaluation

Descriptive statistics comprised frequencies and respective percentages. *χ*^2^ tests were applied to test for differences between groups. Kaplan–Meier plots were used to graphically present patient survival over time after index-CUPs, censoring for another cancer diagnosis than CUP. Twenty-fifth, 50th, and 75th percentiles of survival time were supplemented by respective 95% confidence intervals (95% CI). All statistical analyses were conducted with Stata/IC 13.1 (StataCorp, College Station, TX).

## 3 Results

Within the 2009 to 2010 time frame, 543 patients were identified with at least 1 CUP diagnosis (index-CUP) in Danish hospitals. One patient was excluded as his/her date of death was registered prior to the index-CUP admission date. Thus, 542 patients, all of whom had their data registered in the CR, made up the project cohort. Observations from 27 patients were adjusted as their date of death (obtained from CRS) preceded that of diagnosis (obtained from NPR). Date of death was considered the more reliable of the 2.

### 3.1 Population and cancer registrations

**Figure 1:**
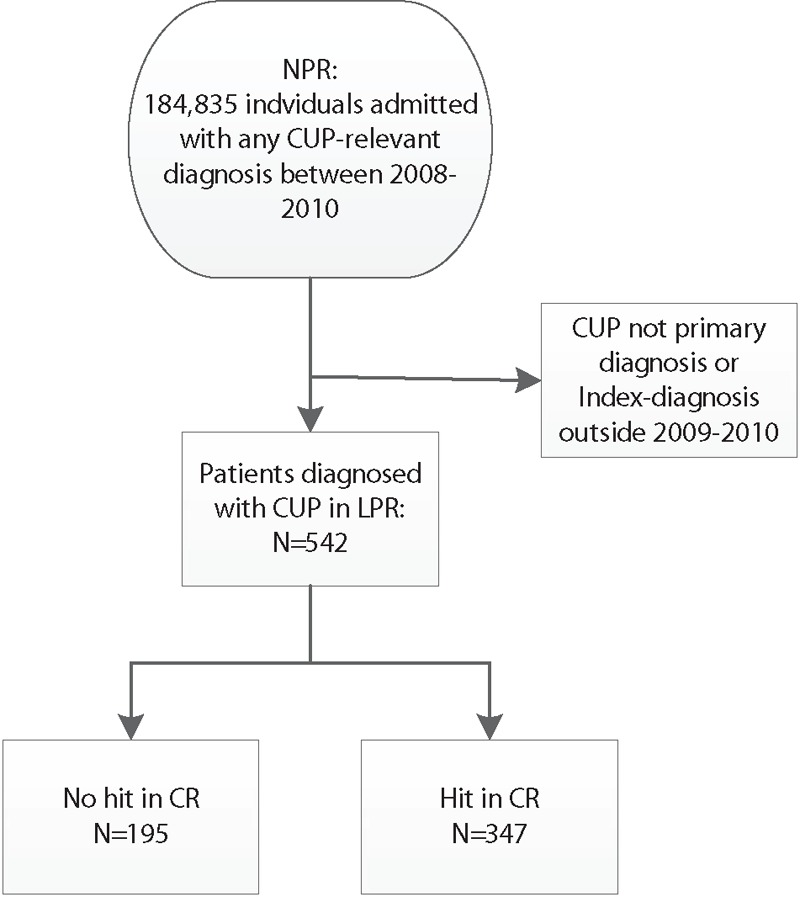
Flowchart of patients. A total of 184,835 patients had any kind of CUP registration in the National Patient Registry (NPR). Restricting the population to relevant registrations decreased the population size to 542 of whom 347 had a hit in Cancer Registry (CR). CUP = cancer of unknown primary.

The 542 patients within the CUP cohort were distributed equally across age ranges and gender with no statistically significant differences (Table 1, see Fig. 1 for flowchart). The first age group included subjects from 0 to 59 so as not to exclude patients. Three patients were younger than 18 years old, that is, 1, 9, and 13 years of age.

**Table 1:**
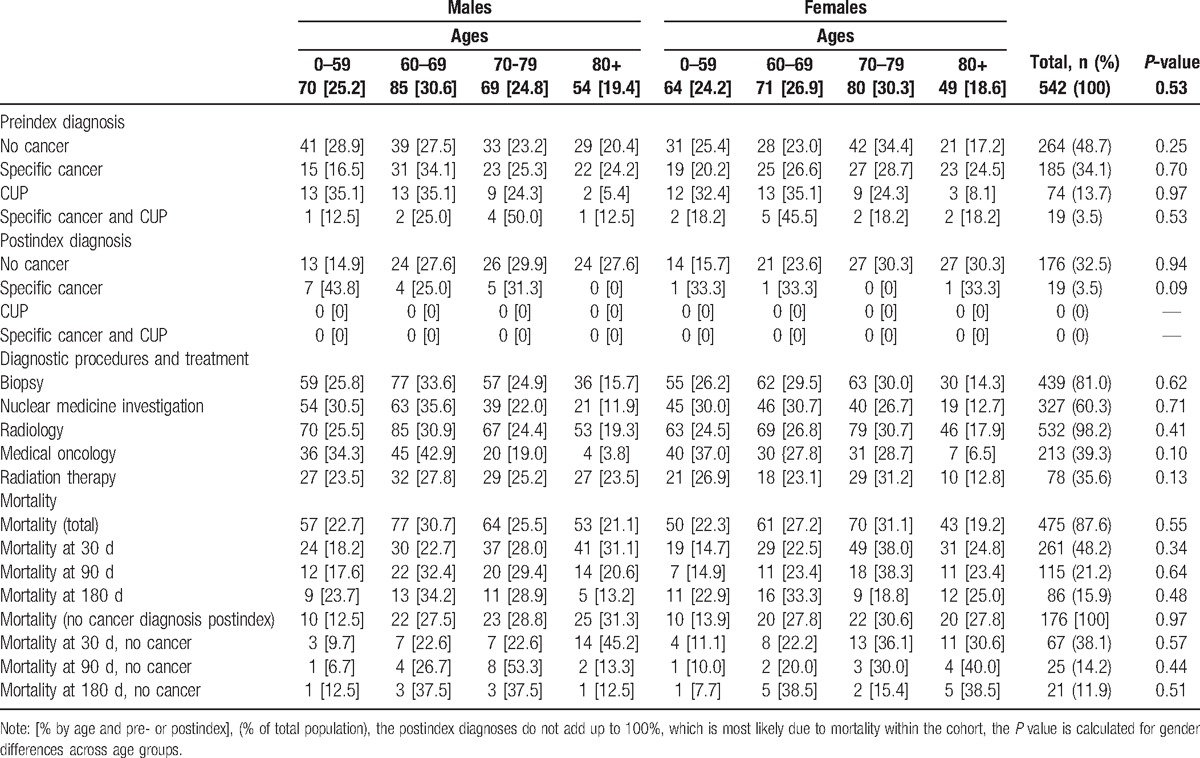
Study population according to gender, age group, diagnoses, mortality, and diagnostic procedures.

A total of 347 patients (64.0%) from the cohort had a hit in CR. A hit was defined as a CR diagnosis within the time interval between 30 days prior to admission until 30 days after discharge as registered in NPR. All patients were, however, registered in CR at some point during their lives. Almost half of the patients had no preindex diagnosis in that they had no cancer registered prior to their index-CUP (n = 264, 48.7%). Approximately one third had a history with a specific cancer (n = 185, 34.1%), and the remaining patients (n = 93, 17.2%) had either a previous CUP diagnosis or both a CUP and a specific cancer (Table 1). Subsequent to the index-CUP, 16 patients had a cancer registered in CR. Among these patients, 10 (2.9%) were registered with a hit and 6 (3.1%) were not registered with a hit (please see Table 2) and thus had postindex diagnoses.

**Table 2:**
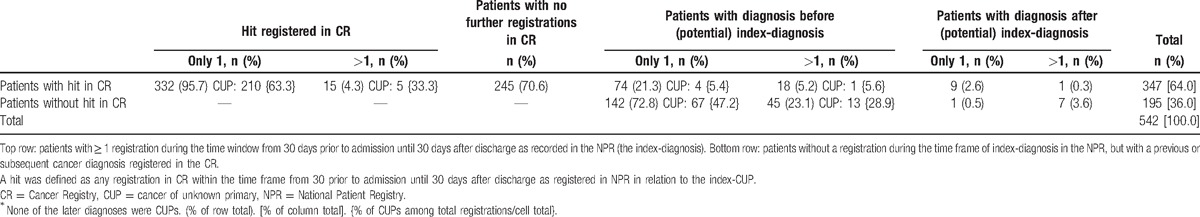
Number of patients with diagnoses registered in the CR.

A total of 439 patients (81.0%) had a biopsy taken, and 532 (98.2%) underwent diagnostic imaging (e.g., computed tomography [CT] and X-ray). Additional imaging within the speciality of nuclear medicine was performed for 327 patients (60.3%). Some patients had procedures registered within medical oncology (n = 213, 39.3%), and 78 (35.6%) received radiation therapy (Table 1).

Between the index-CUP diagnosis and the date of the last observation (July 7, 2013), 475 (87.5%) of the patients had died. Of these, 261 (48.2%) died within 30 days after discharge from the hospital, 115 patients (21.2%) died within 90 days, and 86 (15.9%) within 180 days after discharge. Of the subgroup of patients not diagnosed with a specific cancer subsequent to their index-CUP, 67 (38.1%) died within 30 days, 25 (14.2%) within 90 days, and 21 (11.9%) within 180 days (Table 1). Seven of the 19 patients diagnosed with a specific cancer subsequent to their CUP diagnosis died: 4 within 90 days, 3 between 90 and 180 days (data not shown elsewhere).

### 3.2 Validity of cancer diagnoses at index

When dates of diagnosis from CR and NPR were compared, 195 (36.0%) of patients registered in NPR were not registered with a cancer in CR within a time frame of ± 30 days. Furthermore, 127 patients (23.4%) were registered as having specific cancers, and only 210 (38.7%) were registered in CR as having CUP at the time corresponding to their index-diagnosis in NPR (data not shown elsewhere). The discrepancies between registers indicate that about half of patients who were initially given a CUP diagnosis at hospital admission were later diagnosed with a specific cancer or were not diagnosed with cancer on the basis of diagnostic tests. Diagnoses from CR in conjunction with index-CUP from NPR are presented in Table 2, alongside previous and later diagnoses.

### 3.3 Cancer history

As the CR included data on patients from birth, data were explored for patterns in diagnoses to investigate differences between patients with a hit and patients without a hit. A total of 347 patients (64.0%) had a hit in CR. Among these patients, 74 (21.3%) had 1 previous cancer with 4 being CUPs, and 18 (5.2%) had more than 1 previous cancer 1 of which was a CUP. In comparison, among the patients who did not have a hit in CR 142 (72.8%) had 1 previous cancer with 67 CUPs, and 45 (23.1%) had more than 1 previous cancer including 13 CUPs. These differences are substantial, and may indicate either that a history of cancer is not a predictor for later CUP diagnosis in CR, or patients who have a previous cancer but no hit constitute a group of patients who are not exposed to follow-up investigations due to poor performance status, comorbidity, or other problems that prevent further diagnostics. In terms of diagnoses subsequent to the index-CUP in NPR, only 18 patients were registered. Among these, the patients with a hit accounted for 10 patients (2.9%) with a later diagnosis and 8 patients (4.1%) without a hit. None of the later diagnoses were CUPs (see Table 2).

### 3.4 Clinical guideline compliance

It is not clear from the current dataset to what degree patients had their medical history assessed, or if they were exposed to clinical investigation or blood sampling. In terms of image modalities, however, 532 patients were subjected to at least 1 (first-line) image modality (98.2%). Also, 372 patients (60.3%) had a nuclear medicine procedure (second-line image modality) (Table 1). As many as 439 (81.0%) patients had a biopsy taken. With regard to treatment, 213 patients had registrations within the oncological specialty (39.9%), and 78 had at least 1 registration of radiation therapy (35.6%) (Table 1).

### 3.5 Survival

**Figure 2:**
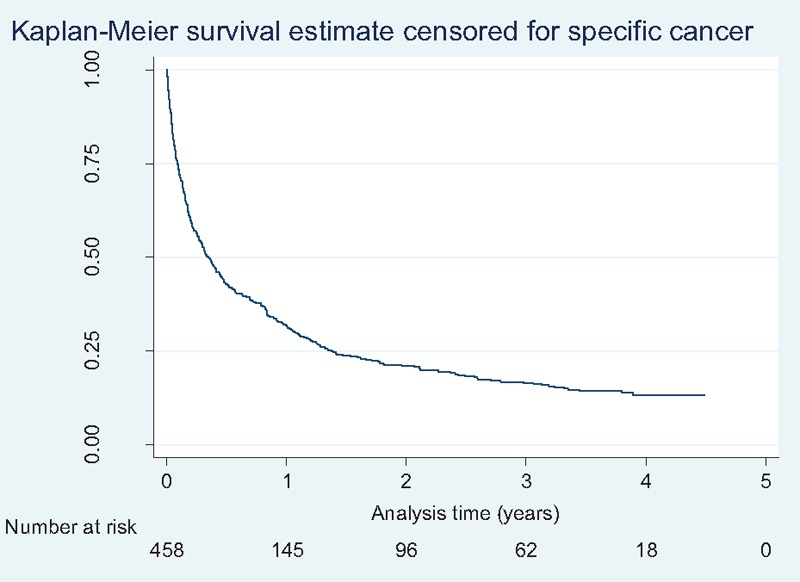
Kaplan–Meier plot of survival. Note: only 458 subjects were considered at risk in the Kaplan–Meier survival estimate at T = 0 because 84 subjects’ date of death was identical to their registered date of discharge.

The prognosis of the patients was poor. The survival curve declined rapidly and steeply (Fig. 2). Median survival from the index-CUP was less than 5 months (129 days, 95% CI: 99–163). One quarter of patients died within 34 days (95% CI: 25–44) and 75% of patients were dead after 503 days (95% CI: 406–717). At the final registration after 4 years, the chance of survival had descended so at the end of study 475 (87.6%) patients had died (Table 1, Fig. 2).

## 4 Discussion

According to the NPR, during 2009 and 2010 a total of 542 patients were diagnosed with CUP at discharge from hospitals in Denmark. The validity of an NPR CUP diagnosis was low, as only 40.6% of the NPR population had their initial diagnosis confirmed. Patients’ histories showed that the most frequent previous cancer was CUP. This finding calls for further exploration in future studies to find a plausible explanation for an apparent lack of consequence of this diagnosis when it was first used, including an analysis of whether the health care system has temporarily lost the patient of sight or found no grounds for further examination and treatment. The low validity of NPR diagnosis and the time span between first (CR) CUP and the index-CUP may not only reflect the work-up challenge for the diagnosis of CUP, but in particular also the difficult situation of the patients and their kin.

The equal distribution of the CUP diagnoses across age and gender was unexpected in view of the typical increase of most cancers with age. Since we could not penetrate deeper into the data, one should be careful to interpret this. However, a speculative possibility could be that cancers in young people are rather uncommon and lack age-related characteristics and therefore perhaps easier downplayed or overlooked. In addition, they are often aggressive and diagnosed at a more advanced stage making detection of the primary more difficult.^[20]^ Albeit rare, some cancer forms are inherited and often present at an early age. Colonic cancers (adenocarcinomas) are less common in young adults but more often linked to a genetic condition that puts the person at higher risk. Thus, they constitute 1 example of malignancies with a high mortality rate among youngsters,^[21]^ particularly so because screening for them is not recommended in young adulthood.^[22]^

Compliance with the work-up part of guidelines was fairly high in that 98.2% of patients underwent diagnostic imaging and 81.0% had a biopsy taken, suggesting that the external validity of results was high, provided that cohort members had been offered the best possible opportunities of identifying their primary. However, only 23.4% of patients had their primary identified at index (according to CR), so despite having undergone recommended diagnostic procedures, only one-fourth of patients had a specific cancer diagnosed. The guideline^[15]^ does not indicate how many primaries should be identified. Thus, it remains unclear to what degree this low number reflects insufficient evidence supporting the guideline.

Survival expectation was low with only 12.4% alive at the end of the study period of 4 years. In CUP, survival may be correlated with insufficient diagnostics in several ways: first, the more specific the diagnosis, the more specific treatment regimens can be instituted, whereas true CUP is treated with more generic chemotherapies of less specificity and, thus, presumably less efficacy in the vast majority of these cancers. Furthermore, the delay in diagnostic pathways due to futile investigations may lead to deterioration of patient performance to such a low status that it contraindicates treatment when the workup is finished. The median survival of 129 days seems to suggest that recommended procedures for diagnosing CUP are insufficient. The confidence intervals of the survival estimates may seem wide; however, perhaps not surprisingly wide considering that the CUP diagnosis covers multiple cancer forms of varying aggressiveness and management, the latter probably ranging from no treatment to aggressive radio-chemotherapy.

### 4.1 Comparison with other research findings

**Figure 3:**
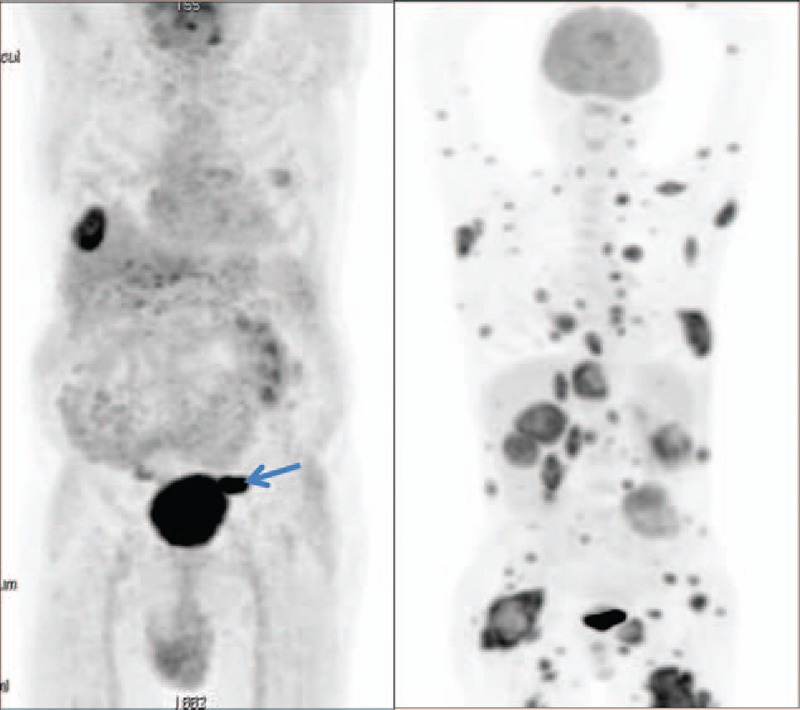
PET/CT in CUP patients. Left: man aged 57 with a biopsy-verified adenocarcinoma metastasis in the liver from a hitherto undetected origin. PET/CT revealed a primary in the sigmoideum (arrow). Right: woman aged 50 with a tumor on her back, diagnosed 4 months later as a metastasis from an unknown primary. PET/CT performed 2 months later revealed multiple metastases precluding identification of the primary. Courtesy: Henrik Petersen, Department of Nuclear Medicine, Odense University Hospital, Denmark.

Epidemiological research in CUP is limited.^[4,23]^ The general consensus in the literature is that CUP is an aggressive cancer with an unfavorable prognosis accounting for a considerable part of malignant cancers.^[4,24]^ The poor survival in our material agrees with data from Sweden, where CUP survival after 1 year was estimated to 20%.^[25]^ Together, these findings call for further research into this cancer in particular.^[1–5]^ Furthermore, research into the modalities used for diagnosis of CUP may potentially change the prognosis of these patients. First, immunohistochemical tumor markers have been mentioned as potentially effective,^[26,27]^ and second, front-line PET/CT has been shown to identify 45% (range 25%–57%) of primary tumors in CUP patients.^[28–31]^ The most recent European Society for Medical Oncology guideline presents a subdivision of CUP cancers on the basis of pathology and recommends physical examination, blood and biochemical analyses, CT, while only recommending PET/CT for a subgroup of patients. The evidence supporting these recommendations is based on retrospective cohorts or case-control studies, or prospective cohort studies.^[27]^ The recommendations are clearly related to references, which is a clear improvement to the Danish guideline. It is not, however, stated how many primaries one can expect to find on the basis of the diagnostic work-updescribed in the guideline. A typical example of delayed PET/CT is shown in Fig. 3.

### 4.2 Strengths and limitations

Our cohort was determined on the basis of NPR. Because of the low validity of this register, our analyses were conducted on patients who did and did not have their CUP confirmed. It is not possible at this point to know how results would have changed if only the group with a valid CUP had been included. Meanwhile, the Danish registries are excellent for research purposes in that they include data on all patients, and that all patients include the entire population as a reference group. Furthermore, the possibility to cross-reference data enables studies that may serve as a basis for future studies and discussions about registration practice. In our study, a CUP diagnosis was considered valid when the diagnostic workup had been performed fully to rule out organ-specific cancers. We were not able to test if our assumption that the CR registrations were actually based on pathologic findings with regard to the CUP diagnosis, which presumably may be more difficult to make than the diagnoses of lung and breast cancer. In future studies, data from the pathology register would serve to further inform the findings.

The date of discharge was used as a reference date for the CUP diagnosis to make sure that all relevant codes of procedures attached to each admission were included in the analyses. Some results may have changed slightly had the date of admission been used instead. For instance, patients are, by definition, alive during their entire admission to hospital, so the survival would have increased somewhat. How much depends on the duration of the admissions, which was not explored here. In line with this, as the duration of admission can vary substantially, it would be interesting in future studies to subdivide diagnostic procedures into 3 time windows, that is, prior to, during, and after admission/discharge.

Only 1 author categorized the codes from NPR into relevance and clinical speciality.

### 4.3 Implications for clinical practice

As this article was based on a cohort and epidemiological methods, the results are of limited immediate value for clinical practice. To inform clinical practice, it would be important for future publications to include an assessment of what diagnostic procedures are most effective in confirming the CUP diagnosis, how early detection may be achieved, and to what degree this may influence the choice and starting point of therapy. Two examples of initiatives facilitating early detection could be the set-up multidisciplinary diagnostic centers^[32]^ or the rapidly evolving field of immunohistochemical markers.^[26]^

### 4.4 Perspectives for future research

Since the data for this project were registered, the diagnostic pathways for CUP (and other cancers) have been updated. Now, an administrative code is applied to all pathways at initiation and termination of the pathway making it possible to identify which procedures have been registered in the diagnostic workup of a specific cancer pathway without making assumptions concerning timing of procedures. Furthermore, 1 aspect that would have informed the current study was which diagnostic cancer pathways the patients had been assigned to. This information was not available to us, but is with the updated codes. In this sense, the current study could be used as a model for updated data collection with the possibility for further explorations as the registration practice has been expanded.

### 4.5 Performance status of patients

Patients’ performance status is of utmost importance in the clinical decision making. As mentioned by Shaw, Adams, Jordan, Crosby,^[10]^ and the Danish Health Authority,^[15]^ a considerable subsection of the CUP population may not have been referred to further diagnostic workup or treatment due to poor general health status. How frequently this is the case can be estimated with the recently introduced administrative codes for cancer diagnostic pathways. In addition, it is uncertain if and after how long time a patient with negative findings in 1 organ-specific pathway is referred to another, in particular because there is no single medical specialty taking care of all suspected CUP patients.

## 5 Conclusion

We found that the validity of CUP diagnoses in NPR is low and that cancer history, and a history of a CUP diagnosis in particular, is relevant as a risk factor for a later CUP diagnosis. Clinicians in Danish hospitals follow the CUP guidelines fairly well in providing recommended diagnostic procedures to most patients, but apparently without improving the survival.

These findings warrant further research focusing on early and more efficient detection of the primary and rapid, targeted treatment, preferably also focusing on the influence of patient performance on the implementation of the recommended diagnostic workup. Danish registries have the potential to serve as tools for these efforts. However, one should not rely on a single register (particularly if that register is NPR). Furthermore, one should ascertain that pathology data are available in all patients.

## Acknowledgment

The authors thank Sonja Wehberg for her support in using Stata for data management.

## Supplementary Material

Supplemental Digital Content

## References

[1] Hemminki K Bevier M Hemminki A Survival in cancer of unknown primary site: population-based analysis by site and histology. Ann Oncol 2012;23:1854–63. 22115926 10.1093/annonc/mdr536

[2] Pavlidis N Pentheroudakis G Cancer of unknown primary site. Lancet 2012;379:1428–35. 22414598 10.1016/S0140-6736(11)61178-1

[3] Pavlidis N Khaled H Gaafar R A mini review on cancer of unknown primary site: a clinical puzzle for the oncologists. J Adv Res 2015;6:375–82. 26257935 10.1016/j.jare.2014.11.007 PMC4522587

[4] Kaaks R Sookthai D Hemminki K Risk factors for cancers of unknown primary site: results from the prospective EPIC cohort. Int J Cancer 2014;135:2475–81. 24692151 10.1002/ijc.28874

[5] Engholm G KA Christensen N The most frequent types of cancer. [Webpage]. 2016; Available at: https://www.cancer.dk/hjaelp-viden/fakta-om-kraeft/kraeft-i-tal/de-hyppigste-kraeftformer/. Accessed May 25, 2016.

[6] Grau C Johansen LV Jakobsen J Cervical lymph node metastases from unknown primary tumours. Results from a national survey by the Danish Society for Head and Neck Oncology. Radiother Oncol 2000;55:121–9. 10799723 10.1016/s0167-8140(00)00172-9

[7] Delgado-Bolton RC Fernandez-Perez C Gonzalez-Mate A Meta-analysis of the performance of 18F-FDG PET in primary tumor detection in unknown primary tumors, Journal of nuclear medicine: official publication. Soc Nucl Med 2003;44:1301–14. 12902422

[8] Varadhachary GR Abbruzzese JL Lenzi R Diagnostic strategies for unknown primary cancer. Cancer 2004;100:1776–85. 15112256 10.1002/cncr.20202

[9] Saliminejad M Bemanian S Ho A The yield and cost of colonoscopy in patients with metastatic cancer of unknown primary. Alim Pharmacol Ther 2013;38:628–33. 10.1111/apt.12429 23869398

[10] Shaw PH Adams R Jordan C A clinical review of the investigation and management of carcinoma of unknown primary in a single cancer network. Clin Oncol 2007;19:87–95. 10.1016/j.clon.2006.09.009 17305260

[11] Pentheroudakis G Golfinopoulos V Pavlidis N Switching benchmarks in cancer of unknown primary: from autopsy to microarray. Eur J Cancer 2007;43:2026–36. 17698346 10.1016/j.ejca.2007.06.023

[12] Lewsey JD Leyland AH Murray GD Using routine data to complement and enhance the results of randomised controlled trials. Health Technol Assess 2000;4:1–55. 11074392

[13] Kaizar EE Estimating treatment effect via simple cross design synthesis. Statist Med 2011;30:2986–3009. 10.1002/sim.4339 21898521

[14] Petersen H Holdgaard PC Madsen PH FDG PET/CT in cancer: comparison of actual use with literature-based recommendations. Eur J Nucl Med Mol Imaging 2016;43:695–706. 26519292 10.1007/s00259-015-3217-0 PMC4764641

[15] Sundhedsstyrelsen. Diagnostic Pathways for Metastases Without Organ Specific Cancer Type [Pakkeforløb for metastaser uden organspecifik kræfttype]. 1.0 ed. København: Sundhedsstyrelsen; 2010.

[16] Friis SJT Mellemkjær L Olsen JH Validation of the National Cancer Registry and Selected Clinical Databases [Validering af Cancerregisteret og udvalgte kliniske cancerdatabaser]. Copenhagen: Kræftens Bekæmpelse i samarbejde med Statens Serum Institut; 2012.

[17] S.K.A. [Co-operative Cancer Departments]. Occult Cancer/Unknown Primary Tumor. Therapeutic Instructions [Cancer occultus/Ukendt primær tumor. Behandlingsvejledning]. 2011.

[18] DAHANCA. Carcinoma metastasis on the neck from unknown primary tumor. Investigation, treatment and follow-up checks. 2013; Available at: file:///C:/Users/ehi6hu/Downloads/Ukendt%20primaertumor%202013_5%20juli.pdf. Accessed June 25, 2016.

[19] Briasoulis E Tolis C Bergh J ESMO Minimum Clinical Recommendations for diagnosis, treatment and follow-up of cancers of unknown primary site (CUP). Ann Oncol 2005;16 Suppl 1:i75–6. 15888766 10.1093/annonc/mdi804

[20] What Are the Key Statistics for Cancers in Young Adults? 2015; Available at: https://www.cancer.org/cancer/cancer-in-young-adults/key-statistics.html#written_by. Accessed 31 March, 2017.

[21] Minardi AJ Jr Sittig KM Zibari GB Colorectal cancer in the young patient. Am Surg 1998;64:849–53. 9731812

[22] American Cancer Society. Cancer Facts & Figures. Atlanta: American Cancer Society; 2016.

[23] Luke C Koczwara B Karapetis C Exploring the epidemiological characteristics of cancers of unknown primary site in an Australian population: implications for research and clinical care. Aust N Z J Public Health 2008;32:383–9. 18782405 10.1111/j.1753-6405.2008.00260.x

[24] Massard C Loriot Y Fizazi K Carcinomas of an unknown primary origin—diagnosis and treatment. Nat Rev Clin Oncol 2011;8:701–10. 22048624 10.1038/nrclinonc.2011.158

[25] Hemminki K Ji J Sundquist J Familial risks in cancer of unknown primary: tracking the primary sites. J Clin Oncol 2011;29:435–40. 21189391 10.1200/JCO.2010.31.5614

[26] Varadhachary GR Raber MN Cancer of unknown primary site. N Engl J Med 2014;371:757–65. 25140961 10.1056/NEJMra1303917

[27] Fizazi K Greco FA Pavlidis N Cancers of unknown primary site: ESMO Clinical Practice Guidelines for diagnosis, treatment and follow-up. Ann Oncol 2015;26 Suppl 5:v133–8. 26314775 10.1093/annonc/mdv305

[28] Nanni C Rubello D Castellucci P Role of 18F-FDG PET-CT imaging for the detection of an unknown primary tumour: preliminary results in 21 patients. Eur J Nucl Med Mol Imaging 2005;32:589–92. 15726356 10.1007/s00259-004-1734-3

[29] Ambrosini V Nanni C Rubello D 18F-FDG PET/CT in the assessment of carcinoma of unknown primary origin. La Radiol Med 2006;111:1146–55. 10.1007/s11547-006-0112-6 17171520

[30] Hu M Zhao W Zhang PL Clinical applications of 18F-fluorodeoxyglucose positron emission tomography/computed tomography in carcinoma of unknown primary. Chin Med J 2011;124:1010–4. 21542959

[31] Han A Xue J Hu M Clinical value of 18F-FDG PET-CT in detecting primary tumor for patients with carcinoma of unknown primary. Cancer Epidemiol 2012;36:470–5. 22504050 10.1016/j.canep.2012.03.002

[32] Sundhedsstyrelsen SoB Diagnostic Pathway for Patients With Non-Specific Symptoms Which Could Be Cancer [Diagnostisk pakkeforløb for patienter med uspecifikke symptomer på alvorlig sygdom, der kunne være kræft]. Vol 2. 0. Copenhagen: Sundhedsstyrelsen; 2012.

