# Supplementary material for: Cancer of unknown primary: Registered procedures compared with national integrated cancer pathway for illuminating external validity
Source: Medicine (Baltimore). 2017 Apr 21;96(16):e6693. doi: 10.1097/MD.0000000000006693 (PMC5406101; doi:10.1097/MD.0000000000006693)
Supplement: Supplemental Digital Content [file medi-96-e6693-s001.docx]

# *Supplementary digital content*

## Data registires

The NPR is structured with a) administrative information, b) diagnoses, c) diagnostic procedures and treatments, and d) surgery. Each contact is identified by a unique record-number. All variables are registered through codes of letters and numbers, which are translatable to text via the so-called SKS-browser^[[1]](#footnote-2)^ (1) and through the documentation of the databases and Statistics Denmark (2).

Data from CR are structured according to tumour-number and the personal ID number that all Danish citizens are provided with at birth. Pseudo-anonymised data from NPR and CR are, upon request, provided with the same person-ID enabling combination of datasets at individual level.

CSR holds data on Danish citizens’ dates of birth and death, which were the only variables from the registry used in this project.

The request for data from NPR, CR, and the CRS had a wide scope to ensure inclusion of all relevant observations. The datasets used in analyses were limited to include only the cohort of patients with their first CUP diagnosis as primary diagnosis registered in NPR during 2009-2010 (from this point on referred to as the index-CUP or index-diagnosis). We decided to identify index-CUPs on the basis of recently developed cancers only. Consequently, NPR data included observations from 2008, although the cohort was initiated by 1 Jan 2009. With this wider population, the inclusion of patients into the cohort was restricted to comprise only patients without registrations of CUP diagnoses in 2008. Secondly, the data from CR included all available retrospective and prospective cancer-history within the population.

## Data definitions

“CUP” was defined as a diagnosis registered as primary in the administrative information of NPR including the codes C768 and C80* indicating “Malignant neoplasms of ill-defined, secondary and unspecified sites” according to the ICD-10 (3). “Valid CUP” meant confirmed by registration in CR.

“Cancer diagnoses” were obtained from CR, as the register includes data on all cancer diagnoses in Denmark. Since the date-correspondence between CR and NPR could not be assumed to be a 100% match, the index-CUPs were identified by allowing a time window of +/- 30 days of the discharge from the index-diagnosis in NPR. Previous cancers were thus determined to be diagnosed >30 days prior to the index-diagnosis (pre-index diagnoses), and later cancers were diagnosed >30 after the index-diagnosis (post-index diagnoses). In case of more than one diagnosis within the +/- 30 days window, the one closest to the date of discharge in NPR was used (17 observations).

“Diagnostic procedures”: One author (AG) manually reviewed all disease and procedure codes within the dataset, matched codes with text explaining the interventions in ICD-10, and grouped all codes according to a) ‘relevance for CUP’ (binary) and b) “medical speciality” (biopsy, medical oncology, nuclear medicine intervention, radiological intervention, and radiation therapy). In addition, the medical specialities including diagnostic imaging were subdivided into first-line and second-line diagnostics imaging according to the clinical guideline. Thus, the first-line diagnostic imaging modalities included X-rays, CT-scans, MRI scans, and ultrasound. The second-line diagnostics included nuclear imaging, i.e. PET/CT.

## CUP guideline

In cases where these standard procedures led to the identification of a primary, the patient should be referred to the organ-specific department. In cases where the preliminary investigations did not identify a primary, the patient would be referred to the oncology department for further diagnostics and/or treatment (4). In the description of responsibilities within the oncological department, the guideline states that the results of patients’ diagnostic procedures should be reassessed and supplemented with a second biopsy. If this turns out to identify the primary, the patient is referred to the relevant medical speciality, and in remaining cases, patients must be offered treatment as soon as possible due to their poor prognosis. As there is no standard treatment, it is recommended that patients are included in trials to make sure that their data are collected systematically for further knowledge generation. Treatment options were described as chemotherapy, radiation therapy, surgery, or a combination thereof (4).

## References

1. SKS-browser, vers 4.02 [database online]. Statens SerumInstitut & National Sundheds-IT; 2016. Updated 2016.04.03.

2. Lynge E, Sandegaard JL, Rebolj M. The Danish National Patient Register. Scandinavian journal of public health 2011;39:30-33

3. WHO. International Classification of Diseases (ICD). 1990. Available at: <http://www.who.int/classifications/icd/en/>. Accessed 2016.05.18, 2016

4. Sundhedsstyrelsen. Pakkeforløb for metastaser uden organspecifik kræfttype. København: Sundhedsstyrelsen; 2010

1. The SKS-browser is an online database that presents all current codes of diseases, treatment, and diagnostic procedures for anyone to look up the explanatory text matching each code. [↑](#footnote-ref-2)
